# Risk factors associated with Crimean-Congo hemorrhagic fever virus circulation among human, livestock and ticks in Mauritania through a one health retrospective study

**DOI:** 10.1186/s12879-023-08779-8

**Published:** 2023-11-06

**Authors:** Abdellahi El Ghassem, Andrea Apolloni, Laurence Vial, Romain Bouvier, Celia Bernard, Mariem Seyidna Khayar, Mariem Cheikh Ahmed, Hugues Fausther-Bovendo, Abdallahi Diambar Beyit, Barry Yahya, Mohamed Bezeid Ould El Mamy, Ahmed Elbara, Mohamed Abdellahi Bollahi, Catherine Cêtre-Sossah, Ali Ould Mohamed Salem Boukhary

**Affiliations:** 1 Université de Nouakchott, UR GEMI, BP 5026, Nouakchott, Mauritania; 2 ONARDEP, BP 167, Nouakchott, Mauritania; 3 CIRAD, UMR ASTRE, Montpellier Cedex, 34398 France; 4 ASTRE, University of Montpellier, CIRAD, INRAe, Montpellier, France; 5 GUARD, Batiscan, QC G0X 1A0 Canada; 6 Department of Microbiology and Immunology, The University of Texas Medical Branch, Galveston, United States; 7 ISET, Rosso, Mauritania; 8 INRSP, BP 695, Nouakchott, Mauritania

**Keywords:** Crimean Congo hemorrhagic Fever, Epidemiology, Mauritania, Antibody prevalence, Genome detection

## Abstract

**Background:**

Crimean Congo hemorrhagic fever (CCHF) is endemic in Southern Mauritania where recurrent outbreaks have been constantly observed since the 1980’s. The present study is the first to assess CCHFV antibodies and RNA in humans.

**Methods:**

A retrospective study was conducted using 263 humans and 1380 domestic animals serum samples, and 282 tick specimens of *Hyalomma* genus collected from 54 settings in 12 provinces across Mauritania. Antibodies targeting CCHF viral nucleoprotein were detected in animal and human sera using double-antigen ELISA. CCHFV specific RNA was detected in human and animal sera as well as tick supernatants using a CCHFV real time RT-PCR kit. Individual characteristics of sampled hosts were collected at the same time and data were geo-referenced. Satellite data of several environmental and climatic factors, were downloaded from publicly available datasets, and combined with data on livestock mobility, animal and human density, road accessibility and individual characteristics to identify possible risk factors for CCHFV spatial distribution. To this end, multivariate logistic models were developed for each host category (human, small and large ruminants).

**Results:**

The overall CCHFV antibody prevalence was 11.8% [95% CI: 8.4–16.3] in humans (17.9% in 2020 and 5.4% in 2021; p = 0.0017) and 33.1% (95% CI: 30.1–36.3) in livestock. CCHFV-specific antibodies were detected in 91 (18.1%) out of 502 sheep, 43 (9.0%) out of 477 goats, 144 (90.5%) out of 161 dromedaries and 179 (74.6%) out of 240 cattle. CCHFV RNA was detected in only 2 (0.7%) sera out of 263 animals herders samples from Hodh El Gharbi province and in 32 (11.3%) out of 282 *Hyalomma* ticks. In humans as well as in animals, seropositivity was not associated with sex or age groups. The multivariate analysis determined the role of different environmental, climatic and anthropic factors in the spatial distribution of the disease with animal mobility and age being identified as risk factors.

**Conclusion:**

Results of the present study demonstrate the potential risk of CCHF for human population in Mauritania primarily those living in rural areas in close vicinity with animals. Future studies should prioritize an integrative human and veterinary approach for better understanding and managing Crimean-Congo hemorrhagic fever.

**Supplementary Information:**

The online version contains supplementary material available at 10.1186/s12879-023-08779-8.

## Background

Crimean-Congo hemorrhagic fever (CCHF) is a tick-borne arboviral zoonotic disease caused by a negative sense single-stranded RNA *Orthonairovirus* named CCHF virus (CCHFV) belonging to the family *Nairoviridae* [1]. Hard ticks of the family *Ixodidae*, and the genus *Hyalomma* are the main vectors of CCHFV. Numerous wild and domestic animals, such as cattle, goats, sheep, camels and hares, act as amplifying hosts for the virus after being bitten by infected ticks, but infection in various animals is mostly asymptomatic [1, 2]. Transmission to humans occurs as a result of being directly bitten by adult infected tick or through direct contact with blood, secretions, or infected tissue from viremic animals (animal-to-human transmission) or patients (human-to-human transmission) [3]. Human to human transmission of CCHFV mostly occurs in health care workers [4–6]. Infected individuals harbor a variety of symptoms, ranging from asymptomatic or mild febrile illness to severe disease characterized by hemorrhagic manifestations, multi-organs failure and shock. The case fatality rate of CCHF ranges from < 5% to approximately 30% among hospitalized patients [5, 7]. Livestock herders and workers, butchers, and slaughterhouse workers in endemic areas are at the highest risk of CCHF infection [4, 8, 9].

CCHF is a widespread zoonosis with reported detection of virus and/or virus-specific antibodies from over 57 countries across Africa, Asia, Europe and the Middle East [7, 10]. In Europe, CCHF is considered an emerging disease [11]. In West Africa, CCHF is endemic in Southern and Southeastern Mauritania and Northern Senegal where sporadic and more recently recurrent outbreaks have been constantly observed since the 1980’s [5, 12–14]. Furthermore, the four most important Mauritanian domestic livestock species (small ruminants, cattle and camels) have been shown to play an important role in the epidemiology of the disease in the country where high level of CCHFV IgG antibody prevalence were recently reported [15]. However, CCHFV in humans has never been investigated despite the recent increase in the number of reported CCHF outbreaks in West Africa and particularly in Mauritania. According to the CCHF world distribution, Mauritania has become one of the countries where an average of 5–49 human cases were reported per year [16] making CCHF as one of the priority zoonotic diseases in Mauritania. The present one health study was the first one to be conducted, including humans, domestic livestock and ticks. The objectives were to provide evidence of CCHFV circulation in Mauritania, determine infection risk areas and associated factors, and update our current knowledge on the epidemiology of CCHF in livestock and humans in Mauritania.

## Materials and methods

### Study area

**Fig. 1:**
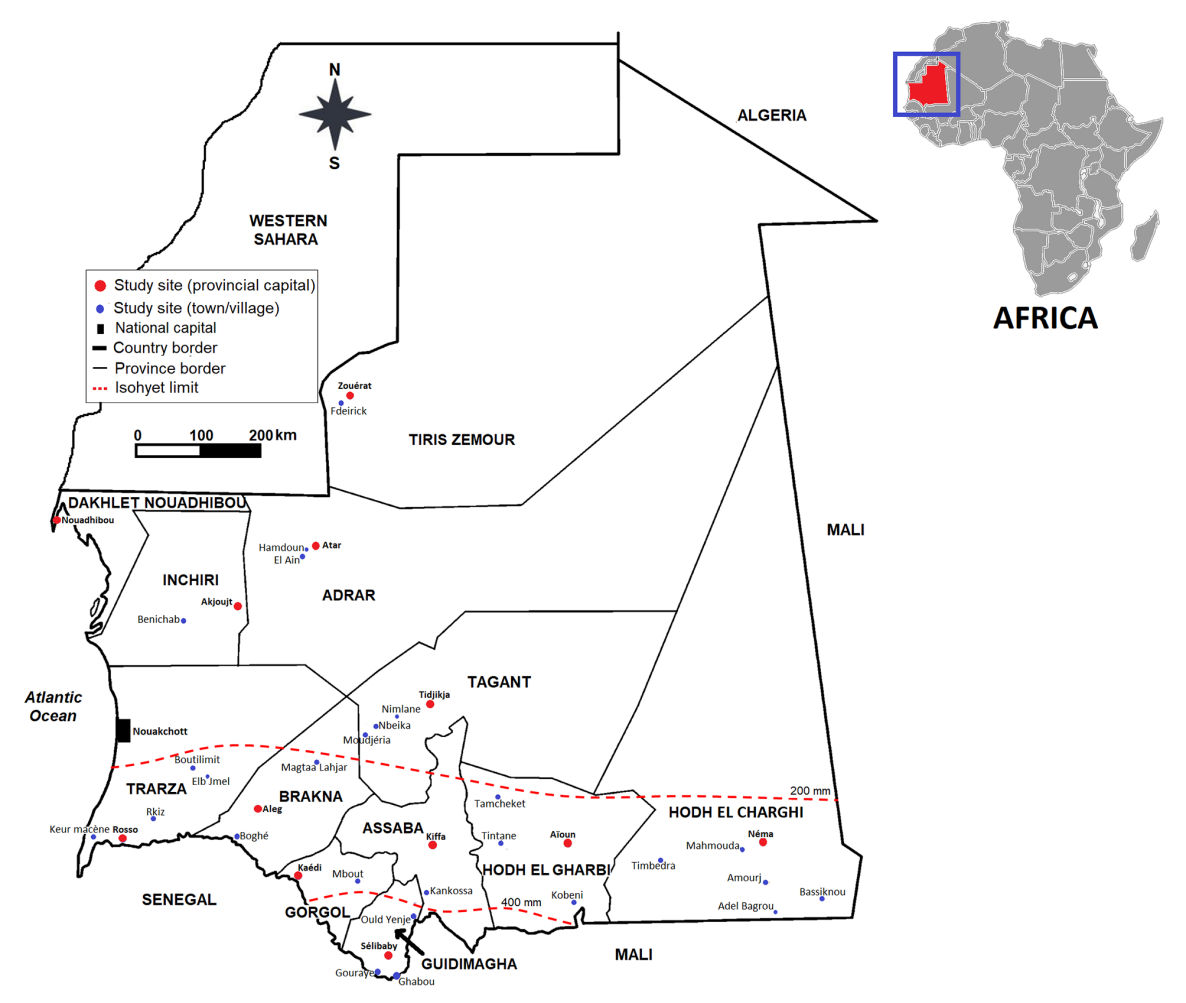
Map of Mauritania showing the study site

The study included 52 study sites distributed across 12 out of the 15 Mauritania administrative provinces (officially known as *Wilayas*) (Fig. 1). The remaining 3 provinces that administratively constitute Nouakchott, the capital city, were not included. Mauritania is located at the confluence between the Sahara and the Sahel spreading over an area of more than one million km². Administratively the country is divided into 15 provinces comprising 63 districts (known as *Mougathaas*) and 220 communes [17]. The country is almost desert, with the Saharan zone covering the northern two-third part of the country, while the remaining southern one-third belongs to the Sahelian zone [18]. According to the aridity index (AI), there were three discrete ecological zones in Mauritania namely the semi-arid zone (0.2 ≤ AI < 0.5) at the southernmost part of the country including the province of Guidimagha and part of Gorgol and Assaba, the arid zone (0.05 ≤ AI < 0.2) included the central provinces of Assaba and Brakna, the southern province of Trarza and the southeastern provinces of Hodh El Garbi and Hodh El Charghi, and the hyper-arid zone (AI < 0.05) comprising the provinces of Tagant, Adrar, Tiris Zemour, Inchiri, Dakhlet Nouadhibou and Nouakchott the capital city [19]. Mauritania has a population of slightly more than 4 millions inhabitants of whom 55% live in urban areas [17], unequally distributed across the country with approximately 2 inhabitants/Km^2^ in the northern Saharan zone against a density of 6 inhabitants/km^2^ in the southern Sahelian zone. The country has a considerable herd of around 2 millions cattle, 1.5 million dromedaries and more than 25 millions small ruminants [20]. Livestock rearing is the main activity in the rural areas and, in the majority of cases, is of the extensive type. Depending on the ecological zones, the greatest number of cattle and sheep are found in the Southern Sahelian zone, while in the Northern Saharan zone, dromedary breeding is the most common livestock practice [20]. Mauritanian population in general and herders in particular traditionally live in close contact with their animals and these herds are most often on perpetual inward (within Mauritania) and outward (from Mauritania to the neighboring countries of Senegal and Mali and *vice-versa*) mobility looking for water and forage resources or to be sold in livestock markets.

### Study period

A retrospective CCHFV serosurvey in Humans and small ruminants (goats and sheep) was conducted using banked sera that were collected during routine surveillance of Rift Valley fever in the wet seasons (August-September) of 2019, 2020 and 2021 where most of the local herds returned to their homelands after months of transhumance. While in large ruminants, banked sera collected during a RVF outbreaks of 2021 and 2022 were used. Ticks survey was conducted only during the dry season of 2019 (February-May) to assess the extent of virus circulation after the 2019 CCHF outbreak in 3 provinces (Hodh El Chargui, Hodh El Gharbi and Trarza) [21].

### Human and livestock serum samples

For human, a total of 263 serum samples (134 in 2020 and 129 in 2021) from healthy participants including 141 slaughterhouse workers and 122 animal herders were selected from the banked sera by simple random sampling. For livestock, a total of 1,380 serum samples including 502 sheep, 477 goats, 161 dromedaries and 240 cattle were randomly selected from the animal banked sera.

### Tick sample collection

A total of 282 ticks collected from livestock in 2019 in areas where CCHF cases have been confirmed, were included in the study. Collected ticks were sorted to different genera using the taxonomic key of Walker et al. [22] and those belonging to the genus *Hyalomma* were included in the study.

### CCHFV antibody detection

Human and animal sera were screened for the presence of total antibodies against CCHFV nucleocapsid protein using the ID Screen^®^ CCHF Double Antigen Multi-species, (IDvet, Grabels, France) according to the manufacturer’s instructions as detailed elsewhere with a 95% CI for sensitivity of 96.8–99.8%, and 95% CI for specificity of 99.8–100% [23].

### Molecular detection of CCHFV in human, livestock and tick samples

Viral RNA was extracted from individual human serum using a semi-automated KingFisher Flex platform (Thermo-Fisher-Scientific, Waltham, MA, USA) and the NucleoMag Vet kit (Macherey-Nagel, Düren, Germany) following the manufacturer’s instructions. Briefly, 100 µL sample volume was added to the KingFisher 96 deep-well plate, followed by the addition of 20 µL Proteinase K and 100 µL lysis buffer VL1. Subsequently, 350 µL binding buffer VEB and 20 µL NucleoMag B-beads were added to the sample-lysis buffer mix. After three washing steps, the extracted nucleic acids were eluted in 100 µL elution buffer VEL and stored at – 80 °C until use. CCHFV RNA detection in human sera was performed using a Crimean-Congo Hemorrhagic Fever Virus Real Time RT-PCR Kit (Liferiver™ Zi Bio-Tech Co., Ltd, Shanghai, China) as recommended by the manufacturer’s instructions. Briefly, 5 µl RNA sample template was added to a 20 µl Master Mix volume reaction comprising 18 µl of Super Mix, 1 µl of Enzyme Mix, and 1 µl Internal Control. The cycling conditions were 45 °C for 10 min, followed by 95 °C for 15 min, then 40 cycles of alternating temperature cycling of 95 °C for 15 s and 60 °C for 1 min.

Extraction of viral RNA from individual animal serum was performed using QIAamp Viral RNA Mini Kit (QIAGEN, Hilden, Germany). Only 141 sera from small ruminants were used to screen for CCHF virus. Screening for CCHF virus was performed using a one-step multiplex qualitative real-time RT PCR developed by Sas et al. [24].

Extraction of viral RNA from individual tick was performed by homogenizing tick in 400 µl of a DNA/RNA Shield buffer (Zymo Research Corp.) and total RNAs was extracted from tick supernatants using a Quick RNA Viral kit (Zymo Research Corp.) according to manufacturer’s instructions. The presence of CCHFV RNA in tick supernatant was determined by qualitative real-time RT PCR protocol developed by Atkinson et al. [25].

### Data analysis

Data were entered into an Excel spreadsheet (Microsoft Office Excel 2007, Microsoft Corporation, Redmond, WA). Proportions were compared using Pearson’s Chi-square test or Fisher’s exact test with a significance level retained for p < 0.05. Univariate analysis was performed and the association between seropositivity and age and sex was assessed using the chi-square test. Statistical significance was defined as a two-tailed p-value < 0.05.

### Multivariate risk analysis

To identify risk factors that may be related to the geographical distribution of CCHF in Mauritania, we developed mixed effect generalized linear models of the individual seroprevalence against individual and spatial factors. For this purpose, we used freely available spatialized databases for environmental (NDVI, Aridity Index), climatic (rainfall, temperature) and anthropogenic (animal and human population, accessibility, distance from road, distance from trade nodes, road, mobility) factors that may be related to tick ecology, as well as to animal driving behaviors. The complete list of factors used is provided in Table 1, supplementary data. The data were provided in spatial raster format and in the case of some climatic factors, time series were provided at regular intervals during the year. For our analysis we considered the mean value within a 10 km radius of the sampling area and in the case of temporal data, we considered the temporal mean and its mean variation interval.

**Table 1:** Number of tested human and animal samples for total anti-CCHFv antibodies and virus detection in different province in Mauritania

Bioclimatic zone	Province	No. study sites (animal, human)	No. of tested animals					No. tested human subjects (%)
			Sheep	Goats	Dromedary	Cattle	Total (%)	
Sahel								
	Assaba	(2, 2)	48	28	30	30	136(14.0)	26(13.2)
	Brakna	(5, 2)	76	29	6	30	135(13.7)	13(6.6)
	Gorgol	(7, 3)	47	42	0	30	119(12.1)	16(8.2)
	Guidimakha	(6, 4)	77	28	8	30	143(14.6)	32(16.3)
	Hodh Echarghi	(5, 2)	76	12	15	30	133(13.5)	43(21.9)
	Hodh Elgharbi	(7, 3)	71	37	15	30	153(15.6)	49(25.0)
	Trarza	(5, 3)	47	58	27	30	162(16.5)	17(8.8)
	Total (%)*		442 (88.1)	234(49.1)	101(62.7)	210(87.5)	987(71.5)	196(74.5)
Sahara								
	Adrar	(4, 1)	19	53	31	NA	103(26.2)	10(14.9)
	Dakhlet Nouadhibou	(1, 1)	18	28	0	NA	46(11.7)	8(11.9)
	Inchiri	(3, 1)	6	52	0	NA	58(14.7)	12(17.9)
	Tagant	(7, 1)	17	73	29	30	149(38.0)	32(47.7)
	Tiris Zemour	(2, 1)	NA	37	0	NA	37(9.4)	5(7.6)
	Total (%)*	(54, 24)	60(11.9)	243(50.9)	60(37.3)	30(12.5)	393 (28.5)	67(25.5)

* Proportions denote the number of animal/human sampled among total tested animals/humans

NA: not applicant, as corresponding animals does not exists in these provinces

### Mobility data

Animal mobility is a fundamental issue in the Mauritanian livestock industry and is thus one of the fundamental levers for dissemination, we have included it in our analysis. We used the data from the survey conducted in 2015 on mobility in Mauritania [26] which contains information for each species on origin and destination, date of movement, number of heads moved and means of transport. We used the data to construct a mobility network whose nodes correspond to the district and the flows between districts are the weighted links. The weight of each link was estimated as the median annual value of all animals moved on the link, all species and all means of transport combined. For the purposes of our analysis, we considered the district of belonging of each sero-sampling location and then evaluated the distance between the centroid of the district and sero-sampling location. The importance of the district was quantified using the centrality indicators of: betweenness, in/out strength and in/out closeness. The correlation between these measures was tested using the Kendall rank test and found to be non-existent. In addition, we studied the structural equivalence of the district and defined clusters as two districts belonging to the same cluster send and/or receive the same number of animals by the other district [27].

### Methodology

For each animal category (small ruminants, large ruminants and humans), we tested the association between CCHF seroprevalence and plausible risk factors in two steps: using the CART (Classification and Regression Tree) technique [28] to identify possible risk factors and categorize them; then testing the association between seroprevalence and the possible factors by multivariate logistic analysis. The CART technique is a non-parametric data mining technique that allows to show relationships between predictors and variable of interest in the form of a hierarchical classification tree. The technique is often used in clinical contexts.

One of the steps of the algorithm is to identify the best variables to divide the outcome into categories, based on the Gini index, and also give an estimate of the importance of the variable. The categories identified by CART were used for univariate logistic analysis and the significance of the association was evaluated by Chi2 test or Cramer’s v value, depending on the sample size. In a second step, we estimated the effect of individual characteristics (Age, Sex, Species/Activity) and environmental, climatic and anthropogenic factors, by informing a Hierarchical Multivariate Logistic Regression Generalized Mixed Effect Model, whose random effect is the sampling locality. Several combinations of predictors have been tested. The “best” model was chosen on the basis of the Akaike Information Criteria (AIC), identifying the models with the lowest AIC value and within a Delta AIC < 2.0, and, in a second step, selecting the models with higher explained deviance rate and higher values of Somers’ Dxy rank correlation and the corresponding AUC for the ROC curve (predictive ability).

For this phase we focused on the 2021 data, year in which seroprevalence data for the 3 categories were collected, which also allowed us to estimate correlations between human and animal serology.

Data processing and analysis was performed using R programming language (version 4.2.1) and the packages: igraph, rpart, Ime 4, MuMIN, ggplot2 and tmap. For spatial dataset the QGIS software has been used for processing the data.

## Results

### Characteristics of the study population

Details regarding the animal species and humans, and geographic origin of the study population are given in Table 1. Overall, 54 study sites for animal survey and 24 study sites for human survey belonging to 12 administrative provinces in the Sahelian (7 provinces) and Saharan (5 provinces) zones across Mauritania were prospected. In the hyper-arid zone, cattle samples came from only the Tagant province at the southern limit of the Sahara as in the remaining northern Saharan provinces cattle rearing is not carried out. The majority of human participants, 74,5% (196/263) were from the Sahelian zone while 25.5% (67/263) were from the Saharan zone (Table 1). Human participant ages ranged from 17 to 78 years with a median (interquartile range) age of 40 (18) years and 259 (98.5%; 95% CI: 95.9–99.5) were males and 4 (1.5%; 95 CI: 1.1 to 10.2) were females.

Animal sampled (n = 1,380) include 502 sheep, 161 camel, 477 goats and 240 cattle of whom 28.5% (393/1380) came from the Saharan zone and 71.5% (987/1380) were from the Sahelian zone (P < 0.0001) (Table 1). The majority, 1282 (90.9%; 95% CI: 90.0-93.7) of animals were females, and the rest, 128 (9.1%; 95% CI: 6.2–9.1), were males. The animal’s median (interquartile range) age were 3 (3) years in Sheep, 3 (3) years in goats, 7.5 (4) years in cattle and 8 (4) years in dromedary.

### Serological study in the human and livestock compartments

Results of the seroprevalence analysis are summarized in Tables 2, 3, 4 and 5. The overall prevalence of anti-CCHFV antibodies was 11.8% [95% CI: 8.4–16.3] in humans (17.9% in 2020 and 5.4% in 2021; P = 0.0017) whereas the overall anti-CCHFV antibody prevalence in livestock was 33.1% (95% CI: 30.1–36.3) detected at 13.7% (95% CI: 11.5–16.2) in small ruminants (18.1% in sheep and 9.0% in goats) and 80.5% (95% CI: 72.0-89.8) in large ruminants (89.4% in dromedaries and 74.6% in cattle) (Table 2). In 2021, where samples from small and large ruminants were obtained, the overall seroprevalence was 47.5% (95% CI: 41.8–53.9) (Table 2). Seroprevalence in small ruminants, significantly increased from 10.8% to 2019 to 19.6% in 2021 (P = 0.0015). Large ruminant also showed a significant increase in seroprevalence from 76.5% to 2021 to 87.9% in 2022 (P = 0.006) although only dromedary was sampled in 2022.

**Table 2:** Seroprevalence of total anti-CCHFv antibodies by study year in human and domestic livestock in Mauritania

		Study year, No. positive/No. tested (%)				
		2019	2020	2021	2022	Total
Human			24/134 (17.9)	7/129 (5.4)		31/263 (11.8)
Small ruminants						
	Sheep	28/205 (13.6)	25/162 (15.4)	38/135 (28.1)		91/502 (18.1)
	Goats	17/209 (8.1)	14/148 (9.4)	12/120 (10.0)		43/477 (9.0)
	Total small ruminants	45/414 (10.8)	39/310 (12.5)	50/255 (19.6)		134/979 (13.7)
Large ruminants						
	Dromedary			20/20 (100.0)	124/141 (87.9)	144/161 (89.4)
	Cattle			179/240 (74.6)		179/240 (74.6)
	Total large ruminants			199/260 (76.5)	124/141 (87.9)	323/401 (80.5)
Total animals				245/515 (47.5)		457/1380 (33.1)

Anti-CCHFV antibodies were found in sera of humans and animal species tested from almost all provinces (Table 3). The highest seroprevalences in humans were found among participants from Dakhlet Nouadhibou province (75.0%; 6/8) followed by those from Hodh El Gharbi province (28.6%; 14/49). Regarding animals, dromedary sera exhibited the highest rates of anti-CCHFV antibodies whatever the province from which they were sampled, with Adrar province (Northern Mauritania) exhibiting the lowest prevalence (77.4%) [95% CI: 59.9–88.9] and Brakna region (Central Mauritania) the highest one (100%; [95% CI: 55.7–100.0]). There were no significant difference between seropositive and seronegative ruminants or humans according to the age groups (Table 3). Regarding the sex of screened ruminants, although proportions of females were significantly higher than males, proportions of seropositive and seronegative animals were often not statistically different whatever the animal species. The same trend was observed in human participants (Table 4).

**Table 3:** Seroprevalence of total anti-CCHFv antibodies in livestock and humans sera from various province in Mauritania

Province	No. positive/No. tested (%)				
	Sheep	Goats	Dromedary	Cattle	Human
Adrar	0/19 (0)	5/53 (9.4)	24/31 (77.4)		0/10 (0)
Assaba	7/48 (14.6)	1/28 (3.6)	25/30 (83.3)	28/30 (93.3)	3/26 (11.5)
Brakna	12/76 (15.8)	1/29 (3.4)	6/6 (100.0)	29/30 (96.6)	1/13 (7.7)
Gorgol	1/47 (2.1)	4/42 (9.5)		24/30 (80.0)	0/16 (0)
Guidimagha	3/77 (3.9)	1/281 (3.6)	8/8 (100.0)	20/30 (66.6)	1/32 (3.1)
Hodh Echarghi	21/76 (27.6)	1/12 (8.3)	14/15 (93.3)	20/30 (66.6)	2/43 (4.6)
Hodh Elgharbi	17/71 (30.0)	8/37 (12.6)	14/15 (93.3)	29/30 (96.6)	14/49 (28.6)
Inchiri	0/6 (0)	1/52 (1.9)			0/12 (0)
Dakhlet Nouadhibou	11/18 (61.1)	5/28 (17.8)			6/8 (75.0)
Tagant	1/17 (5.9)	4/73 (5.5)	27/29 (93.1)	13/30 (43.3)	1/32 (3.1)
Tiris Zemour		1/37 (2.7)	29/30 (96.6)		0/5 (0)
Trarza	18/47 (38.3)	11/58 (18.9)	26/27 (96.3)	16/30 (53.3)	3/17 (17.6)
Total	91/502 (18.1)	43/477 (9.0)	144/161 (89.4)	179/240 (74.6)	31/263 (11.8)

**Table 4:** Seroprevalence of total anti-CCHFv antibodies in human and livestock sera according to age groups in Mauritania

		Age groups (year)	No. tested	No. positive (%)	*p-*value
Human		17–31	75	7 (9.3)	0.25
		32–39	59	5 (8.5)	
		40–49	65	7 (10.7)	
		> 50	64	12 (18.7)	
Small ruminants					
	Sheep	1–2	166	25 (15)	0.08
		3–4	201	33 (16)	
		5–10	135	33 (24)	
	Goats	1–2	217	19 (8.7)	0.93
		3–4	188	18 (9.6)	
		5–9	72	6 (8.3)	
Large ruminants					
	Dromedary	1–2	11	11 (100)	0.28
		3–5	27	26 (96.3)	
		6–9	61	55 (90.1)	
		10–20	62	52 (83.8)	
	Cattle	1–2	10	5 (50)	0.27
		3–5	35	27 (77.1)	
		6–9	134	103 (76.8)	
		10–16	61	44 (72.1)	

### Genome detection of CCHFV in humans, animals and ticks

Of 263 human sera tested for the presence of CCHFV RNA, 2 samples (0.7%) from animal herders in Hodh El Gharbi were positive. The stock of reagents was insufficient to test all animal serum samples. Thus, only 141 animal sera randomly selected were tested of which none were positive. Of 282 ticks included in the study, CCHFV RNA was detected in 32 (11.3%) of them (data not shown). There were 23.4% (22/94) positive ticks collected on sheep, 5.3% (7/131) collected from cattle, and 5.2% (3/57) collected on camels. No positive tick was collected from goats. Positive *Hyalomma* ticks were collected from Bassiknou (3/94, 3.2%), in the Hodh El Chargui province, south-eastern arid zone of Mauritania, Tintane (7/94, 7.4%), in the Hodh El Gharbi province, south-eastern arid zone of Mauritania and Nouadhibou (22/94, 23.4%) in Dakhlet Nouadhibou province, northern hyper-arid zone.

### Multivariate logistic model

#### Mobility data

**Fig. 2:**
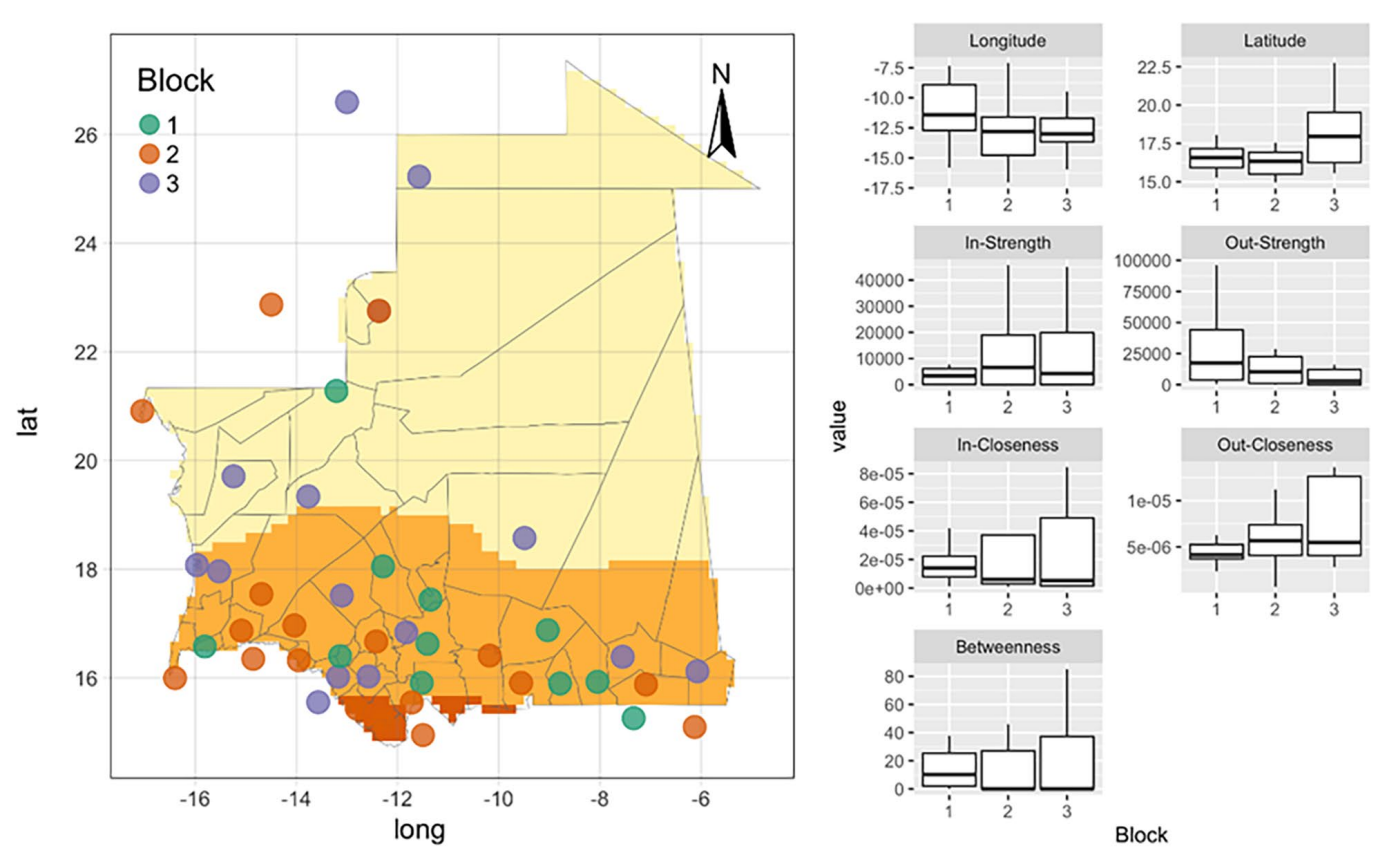
**On the left**: map of the locations involved in the mobility survey. Nodes belonging to same block, i.e. cluster of structurally equivalent nodes, are colored in the same way. Three clusters have been identified in the preliminary network analysis. **On the right**: boxplots for the geographical (longitude and latitude) and network centrality measures (in-strength, out-strength, In-closeness, out-closeness, betweenness) distribution for each block (i.e. a whisker box for each block)

The structural equivalence analysis shows the existence of 3 clusters (called Block). The network characteristics of the Blocks and the geographical distribution are shown in Fig. 2. Block 2 is the core of the network, while very few links exist between Block 1 and 3. Moreover the interaction between Block 1 and Block 2 is much stronger than the interaction between 2 and 3. The nodes of Block 2 are the origins and destination of the movements to/from the Senegal and are mostly concentrated in the southern part of Mauritania, while the nodes of the Block 1 are the origins of national movements. Block 3, on the other hand, consist of nodes on the 2 main national axes (North-South and West-Nouakchott). From a structural point of view, the nodes of Block 1 are moderately more central (high average betweenness), potentially can be affected by disease at the beginning of epidemics (high in-closeness) and potentially be super-spreaders (high out strength). Block 2 and 3 nodes play almost similar roles, with Block 3 nodes attracting more animals (high in-strength) than exporting.

### Categorization of risk factors

We used the CART method to categorize environmental climatic and anthropic factors. In Fig. 1_Supplementary data we show the CART for seroprevalence in large and small ruminants. For each species, the tree shows the different possible splitting rules that can be used to efficiently predict the type of result (seropositive or seronegative). Each box (decision node) corresponds to a single input predictor variable and a split threshold on that variable. The values in the box correspond to the outcome and frequency of the outcome after each cut-off. The different cut-off values allow us to define categories that can be used for multivariate analyses.

In the case of large ruminants, seroprevalence appears to depend on the type of vegetation cover (NDVI), the age and sex of the species, and the temperature: the risk of seroprevalence is high among male animals living in areas with an NDVI between 0.13 and 0.21, and the risk increases with age (> 4 years) or in temperate areas; where the NDVI is higher than 0.21, however, age is a determining factor, and seropositivity is higher for animals older than 4 years; in drier areas (NDVI < 0.13), however, females aged around 5 years appear to have higher seropositivity. For small ruminants, longitude, distance from the movement location (district), age, sex and species appear to have an influence on the seropositivity of the animals. For human hosts, however, the algorithm did not find any group with significantly different seroprevalence.

We used the information from the carts to categorize the continuous variables in the predictors. When the CART for one species did not provide a precise split, we used the split for the other species. We categorized the ground temperature into two categories, low and high, depending on whether it is lower or higher than 34. Similarly, we categorized the NDVI into 3 categories (low < 0.13, High > 0.21, Medium otherwise) and also the longitude we identified 3 zones (East, Center, West) where the Center corresponds to the zone with longitude between − 14 and − 10. The age has been classified in different ways by the 3 categories.

Since the CART for humans didn’t identify any possible cuts, we used categorical variables as provided and quartiles for continuous variables.

### Model results

For each host category (humans, small, large ruminants) we informed a multivariate logistic regression model with the province of locality of sampling as random effect. Secondly, we developed a first model using as risk factors only the individual ones (age, sex, species) and verify that the model improves the prediction. Once the individual factors were identified we used a forward backward procedure adding environmental, climatic and anthropogenic factors to improve the model. In the models we have considered both continuous and categorical variables. Moreover, for continuous variables, like age, environmental indicators, we tested models using either variables in the original form or in the categorical one. The choice of the “best model” was based on the AIC value, but also on the percentage of variance explained and Somers Dxy rank correlation test (predictive ability) and the corresponding AUC for the ROC curve (explanatory ability). The Table 2, supplementary data reports, for each host category, the null model, the model with individual indicators and the best model with their indicators. For categorical variables, coefficients represent the expected variation of the log odds of having an outcome with the respect to a reference category, while for continuous ones, the expected variation in log odds of having outcomes per unit change.

### Large ruminants

**Fig. 3:**
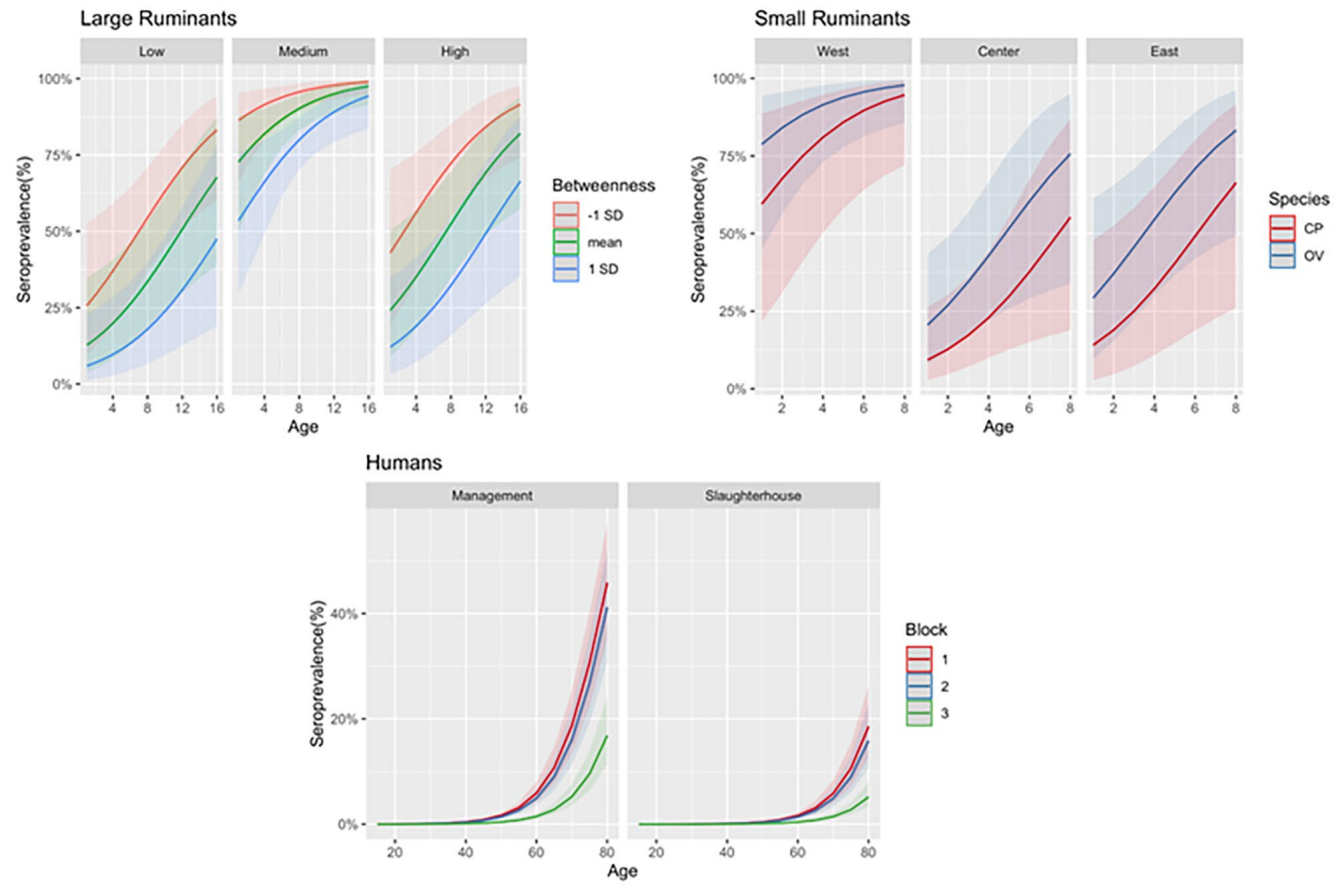
Model prediction, on the x-axis the age and on the y-axis the seroprevalence. For large ruminants, each facet corresponds to NDVI categories. Color corresponds to the Betweenness values (Mean and +/- 1 standard deviation) and shaded areas correspond to 95% Confidence Interval (C.I.) For small ruminants, each facet corresponds to Longitude categories. Color corresponds of the species involved (Mean and +/- 1 standard deviation) and shaded areas correspond to 95% C.I. For humans, each facet corresponds to the type of activity. Color corresponds to the Block the location belongs to and shaded areas correspond to 95% C.I

Results are shown in Table 6, while prediction of the model are shown in Fig. 3. Who shows the predicted behaviour of seroprevalence as function of 3 predictors (Age, NDVI class, and District betweenness), summarizing over all the other predictors (shaded area). Seroprevalence increases with age and with livestock density (Cattle). The betweenness has a protective effect, the localities linked to less central District have a higher seroprevalence. The vegetation cover (NDVI) has a non-linear effect, which increases in the first two classes to learn down for too high NDVI. The serology is higher at all age in areas belonging to the Middle NDVI area. The behavior of serology per age in the Low and High NDVI classes is similar, but serology is slightly higher in the high NDVI area. Most likely this behavior is related to the fact that most of the sampling come from Arid (middle) areas of Mauritania. Centrality in the mobility network (i.e. betweenness) has a similar and important effect in low and high NDVI areas, decreasing with the larger betweenness. Nevertheless, for older animals in Middle NDVI areas the effect is strongly reduced. Sex and species did not have a significant effect.

**Table 5:** Seroprevalence of total anti-CCHFv antibodies in human and livestock sera according to sex in Mauritania

		Sex	No. tested	No. positive (%)	*p-*value
Human		Male	259	30 (11.6)	0.39
		Female	4	1 (25.0)	
Small ruminants					
	Sheep	Male	35	9 (23.1)	0.25
		Female	467	82 (17.5)	
	Goats	Male	20	1 (5.0)	1.00
		Female	457	42 (9.2)	
Large ruminants					
	Dromedary	Male	41	38 (92.6)	0.43
		Female	120	106 (88.3)	
	Cattle	Male	5	5 (100.0)	0.33
		Female	235	174 (74.0)	

**Table 6:** Results of multivariate logistic model for each category

	Large ruminants			Small ruminants			Human		
*Predictors*	*Odds Ratios*	*CI*	*p*	*Odds Ratios*	*CI*	*p*	*Odds Ratios*	*CI*	*p*
(Intercept)	0.11	0.02–0.50	**0.004**	1.03	0.16–6.53	0.975	0.00	0.00–0.00	**< 0.001**
Age	1.19	1.05–1.35	**0.006**	1.43	1.05–1.94	**0.022**	1.14	1.13–1.15	**< 0.001**
Betweenness	0.09	0.02–0.33	**< 0.001**						
Cattle	4.99	1.34–18.62	**0.017**						
NDVI class [Medium]	18.25	5.94–56.10	**< 0.001**						
NDVI class [High]	2.18	0.72–6.62	0.168						
Sex [M]	1091661667.29	0.00 - Inf	0.998						
Species [CMLS]	666779588.14	0.00 - Inf	0.997						
Long class [Center]				0.07	0.02–0.19	**< 0.001**			
Long class [East]				0.11	0.02–0.62	**0.012**			
Dist2 [2.8–78]				0.10	0.03–0.35	**< 0.001**			
Dist2 [79–94]				0.91	0.21–4.02	0.901			
Species [OV]				2.52	1.02–6.22	**0.045**			
Block [2]							0.83	0.82–0.83	**< 0.001**
Block [3]							0.24	0.24–0.24	**< 0.001**
Type [Slaughterhouse]							0.27	0.27–0.27	**< 0.001**

### Small ruminants

Results are shown in Table 6, while prediction of the model are shown in Fig. 3. Who shows the predicted behavior of seroprevalence as function of 3 predictors (Age, Longitude, and Species), summarizing over all the other predictors (shaded area).

Seroprevalence increases with age and is higher in sheep than in goats. There is a gradient of seroprevalence from east to west, with little difference between the central and eastern zones. Nevertheless, the distance from the district also plays an important role, whit seroprevalence the highest in the mobility location.

#### Humans

Results are shown in Table 6, while prediction of the model are shown in Fig. 3. Who shows the predicted behavior of seroprevalence as function of 3 predictors (Age, Activity type, and Block), summarizing over all the other predictors (shaded area).

There is a net difference between the different activities, with seroprevalence higher among those working in herd management. Seroprevalence increases with age, independently of the type of activity and position. The seroprevalence is highest in the areas belonging to Block 1, and is strongly lower in Block 3.

## Discussion

Integrating human, livestock and tick studies in assessing the epidemiology of CCHFV is the keystone for better understanding and managing this zoonotic disease [29]. Livestock species particularly ruminants play a critical role in the dynamic of CCHFV epidemiology by entertaining ticks with the potential to maintain and transmit CCFHV and also by acting as efficient amplifier hosts [29, 30]. The present countrywide survey, demonstrated for the first time the widespread circulation of CCHFV among slaughterhouse workers and animal herders across Mauritania with an average seroprevalence of 11.8% for the period of 2020–2021. Furthermore, high anti-CCHFV antibodies prevalence of 89.4% and 74.6% were detected among domestic dromedary and cattle, respectively. A study conducted by Schulz et al. [15] reported lower seroprevalences among dromedary (81%) and cattle (69%). A lower seroprevalence (67%) was also reported in Mauritanian cattle by Sas et al. [31]. In the neighboring country of Mali, a seroprevalence of 66% was reported in cattle [32]. Regarding dromedary, there were no data from the neighboring southern countries of Mali and Senegal as dromedary breeding is not common in these countries, nor from the northern Maghreb countries like Morocco and Algeria where presence of CCHV is still not reported. However, studies from Tunisia, Sudan and Egypt reported seroprevalences of 89.7%, 21.3% and 14% respectively [33–35].

The high seroprevalence observed among dromedary and cattle could be explained by the longevity of these animals as compared with small ruminants and the persistence of antibodies [13]. Furthermore, these two domestic animal species have been described as suitable hosts for a large number of tick species [36]. In addition, their movement, often over a long distance, play an important epidemiological role in introducing and spreading tick vector and CCHF virus [37].

In small domestic ruminants, CCHFV specific antibody prevalence in sheep (18.1%) was twice more than in goats (9.0%). A previous study conducted in various regions of Mauritania, reported equal seroprevalences (about 15%) in both sheep and goats [38]. In Senegal, a seroprevalence of 38.4% was reported in 34 sentinel sheep during a study conducted in Northeastern Senegal near the Mauritanian border [39]. In another study from Senegal, the seroprevalence of CCHF infection among cattle, sheep and goats was 57.1%, 22.1% and 6.9%, respectively [13]. The difference in seroprevalences between sheep and goats reported in the present survey could be explained by the fact that sheep are more abundant than goats particularly in the southern Sahelian zone. Moreover, sheep were generally kept close together in fences, which facilitate the circulation/dissemination of ticks as pathogens vector among them while goats are left outside. Hence, they were at lower risk of disease. Human overall antibody prevalence in the present survey was 11.8%, comparable to the 10.4% and 13.1% reported from Senegal [40, 41].

During this survey, there were no association between seropositivity within different animal species and humans and the sex or the age groups. This result contrasted with a previous finding by Schulz et al. [15] who showed that seroprevalence in cattle, dromedary, sheep and goats was directly linked to the age of the animals, i.e. older animals had significantly higher seroprevalence rates than younger animals. In our study, although older animal particularly in sheep and cattle and older human subjects showed higher seroprevalences than those observed in younger animals or human subjects, however proportions were not statistically different. Regarding the seroprevalence with respect to the sex of animals, our finding were similar to those already reported by Schulz et al. [15] in Mauritanian livestock. It is worth noting that in the present survey, there were a disproportionately high number of females in animal and human sampled than males, which could have resulted from a selection bias [41]. The high number of sampled animal female is due to the fact that farmers kept females for a longer period than males for breeding purposes aside from the production of milk before being sold on market for meat. In human subjects, the high number of males is due to the fact that herding and slaughtering animals are an exclusively male activities in Mauritania as well as in the Sahel region.

The present study revealed the presence of CCHFV RNA in 0.7% of tested human sera (2 positive human sera out of 263 tested) from Hodh El Gharbi province and its absence in animals sera suggesting possible active circulation of the virus in the study site. However, at this stage and given that only 10% of total animal sera have been tested for presence of CCHFV RNA, a solid conclusion cannot be drawn at present. CCHFV RNA was also found in *Hyalomma* tick populations among domestic livestock with a positivity rate of 11.3%. Unfortunately, collected tick specimens were not identified at the species level. However, a previous study identified the presence of at least 3 species among domestic livestock in Mauritania, including *H. dromedarii*, *H. impeltatum* and *H. rufipes* [38]. In that study, 2.5% of *Hyalomma* ticks collected from camels and cattle tested positive for CCHFV. Furthermore, CCHFV was also detected in neighboring countries, in *H. aegyptium* ticks collected from tortoises in Algeria [42], and in *H. marginatum* ticks collected from birds in Morocco [43]. The CCHFV prevalence observed in ticks collected in epidemiological investigations indicate the importance of investigation conducted out after each case-patient confirmation.

The multivariate analysis has shown the role of different environmental, climatic and anthropic factors in the spatial distribution of the disease. For all the categories, age has appeared always as a risk factor, and the seroprevalence is increasing with age. This was expected since older subjects are exposed for longer time to the threat of CCHF. However, other factors affect the age profiles. Species has been found an important factor for small ruminants with sheep more susceptible than goats, while for large ruminants, due to the large percentage of camels sampled, this does not appear as a relevant risk factor. Individuals working in strict contact with animals (management) appear to be at higher risk of infection. Environmental conditions affect the distribution of CCHF among large ruminants. However, the difference could be related to the sampling procedure. Anthropic factors, appear to play an important role too. In the case of small ruminants, the fact that seroprevalence is higher in the west and the east areas could be related to movement for consumption of meat: in the more inhabited areas (western) animals are brought from abroad (eastern area) and gathered in the area before being killed. The role of mobility is clearer for large ruminants (in particular camels) and humans. More secluded area (low betweenness) can be identified with area at the international borders where CCHF can be introduced due to transhumance movements from abroad. Similarly, for humans we notice that mobility (Block) has a huge impact of seroprevalence. The seroprevalence is high and patterns similar in Blocks 1 and 2. The two blocks are strongly connected among them, almost forming a unique cluster, and could explain the similarity in seroprevalence patterns. For nodes in Blocks 1 and 2 CCHF could be introduced because of international/transhumant movements of small ruminants and cattle to/from Senegal and then diffuse due to local national trades. For locations belonging to Block 3 CCHF could be introduced from the North through camel movements.

CCHF is a one of the priority endemic zoonotic diseases of public health concern in Mauritania [16]. Several factors sustain the transmission and spread of this emerging disease including the great livestock diversity composed of a variety of small and large ruminants, their number estimated at 30,000,000. Ecological conditions also contribute in sustaining CCHFV circulation. Indeed, with a vast northern Saharan arid zone and a southern Sahelian zone characterized by low and heterogeneously distributed amount of rainfall, transhumance mode of pastoralism is prevailing in Mauritania. Consequently, intense inward (within Mauritania) and outwards (from-and-to Mauritania) livestock mobility for grazing constantly occurs [44]. The role of livestock movement in the spread of infectious diseases is well established [45]. In addition to the transhumance movement, livestock is moved to be sold in markets in order to cover the farmers familial needs, or during religious feasts like Tabaski.

From a public health point of view, CCHF outbreaks continue to cause fatality particularly among animal breeders [14]. The geographic distribution of the disease resulted from the present study suggest the presence of the virus among humans, livestock and ticks from almost all prospected provinces in Mauritania. Historically, CCHF first occurred in the extreme southern province of Guidimagha where the first human case in West Africa was detected [12]. Twenty years later, CCHF cases were reported for the first time among human cases in Nouakchott, in the Saharan region, which was considered free from CCHF [5]. The overall fatality rate in that CCHF outbreak was 28.6%. More recently, in 2022, a widespread CCHF outbreak occurred with 7 confirmed cases and 2 deaths highlighting CCHFV circulation (El Bara personal communication). To our knowledge this the first CCHF survey in humans in Mauritania.

Limitations of the present study include absence of CCHFV whole genome sequencing to confirm presence of CCHFV RNA and determine to which virus lineages it belong although previous studies showed the presence of CCHFV genotypes (Africa I and III) either alone of circulating together [39]. Moreover, identification of *Hyalomma* ticks to species level was not performed during this study as collected tick specimens were immediately used in virus detection.

## Conclusion

The present countrywide survey demonstrates a widespread circulation of CCHFV among domestic livestock across Mauritania indicating a potential risk to the human population particularly those involved in the livestock value chain. Moreover, this study has hinted at the role that livestock mobility could play in the circulation of CCHFV. Dromedary, cattle and sheep exhibit a high potential to maintain and transmit CCFHV. As 44% of the human population in Mauritania live in rural areas in close proximity to animals, there is an urgent need for educational programs to increase awareness of CCHF in these communities as well as in general population. In addition, future studies should prioritize an integrative human and veterinary approach for better understanding and managing Crimean-Congo hemorrhagic fever. Databases generated during this survey will be used to assess the risk factors that may be related to the spatial distribution of CCHF antibody prevalence in Mauritania.

### Electronic supplementary material

Below is the link to the electronic supplementary material.

Supplementary Material 1

Supplementary Material 2

Supplementary Material 3

## Data Availability

All data generated during this study are within the manuscript.
