# Supplementary material for: Risk factors associated with Crimean-Congo hemorrhagic fever virus circulation among human, livestock and ticks in Mauritania through a one health retrospective study
Source: BMC Infect Dis. 2023 Nov 6;23:764. doi: 10.1186/s12879-023-08779-8 (PMC10626674; doi:10.1186/s12879-023-08779-8)
Supplement: Supplementary file 1 — Supplementary Material 1 [file 12879_2023_8779_MOESM1_ESM.docx]

Supplementary Table 1: List of predictors used in the multivariate analysis of risk factors

| Parameter | Factors (variable name) | Description | Reference |
| --- | --- | --- | --- |
| Climatic factors | Average annual rainfall and range (Rain) | Raster: accumulated water that falls on Earth surface every 10 days | [1] |
|  | Average annual temperature at 2m (Temp) | Raster : Temperature of the air at 2m above the surface every month | [1] |
| Environmental Factors | Average annual Normalized Difference Vegetation Index (NDVI) | Raster : estimate of the greenness of the vegetation production every 10 days | [2] |
|  | Aridity Index value (Aridity index) | Raster of the aridity Index estimated as the ratio between the annual precipitation and the annual evapopration | [3] |
|  | Ticks Presence (TickP) | Binay value (1 present 0 absent) indicating the presence or not of the competent Tick species in a Moughataa | [4] |
| Anthropic factors | Cattle Population (Cattle) | Raster : cattle heads number by pixel | [5] |
|  | Small Ruminant Population (PR) | Raster : heads number by pixel obtained summing goats and sheep population |  |
|  | Human Population (HH) | Raster: Population by pixel | [6] |
|  | Accessibility (access) | Raster travel time (in minutes) to nearest city 2015 | [7] |
|  | Distance to road (DistRoad) | Crowfly distance to the closest main road | [8] |
|  | Distance to mobility network(Dist) | Crow fly distance from prelevement location to the centroids of Moughataa | [9] |
|  | In/out degree (in/outdeg) In/Out Closeness (in/out close) Betweenness (betw) | Network centrality measures estimated from mobility network | [10] |
|  | Strucutural equivalence Cluster( Block) | Factor (1,2,3,) indicating to which cluster the node belongs | [10] |

References :

1. Muñoz Sabater, J. (2019): ERA5-Land monthly averaged data from 1950 to present. Copernicus Climate Change Service (C3S) Climate Data Store (CDS). DOI: [10.24381/cds.68d2bb30](https://doi.org/10.24381/cds.68d2bb30) (Accessed on 27-04-2023)
2. Copernicus Global Land Service. Provindin bio-geophysical products of global land surface. URL: <https://land.copernicus.vgt.vito.be/PDF/portal/Application.html#Browse;Root=513186;Collection=1000322;Time=NORMAL,NORMAL,1,JANUARY,2020,31,DECEMBER,2020;isReserved=false> (Accessed on 27-04-2023)
3. CGIARCSI. Global Aridity and PET Database. URL: <https://cgiarcsi.community/data/global-aridity-and-pet-database/> (Accessed on 27-04-2023)
4. Gridded Livestock of the Word – 2010 (GLW 3). Université Libre de Bruxelles/Food and Agriculture Organisation. 2018; URL: <https://dataverse.harvard.edu/dataverse/glw_3> (Accessed on 27-04-2023)
5. Morel 1958
6. GDX. Mauritania – Population Counts. URL: <https://data.humdata.org/dataset/worldpop-population-counts-for-mauritania> (Accessed on 27-04-2023)
7. MAP. <https://data.malariaatlas.org/trends?year=2020&metricGroup=Malaria&geographicLevel=admin0&metricSubcategory=Pf&metricType=rate&metricName=incidence> (Accessed on 27-04-2023)
8. <https://geonode.wfp.org/layers/geonode:mrt_trs_roads_osm/metadata_detail>. (Accessed on 27-04-2023)
9. Apolloni A, Nicolas G, Coste C, El Mamy AB, Yahya B, El Arbi AS et al. Towards the description of livestock mobility in Sahelian Africa: Some results from a survey in Mauritania. PLoS One. 2018;24:13(1):e0191565.
10. Selim, A. et al. (2021) 'Determination of Seroprevalence of Contagious Caprine Pleuropneumonia and Associated Risk Factors in Goats and Sheep Using Classification and Regression Tree', Animals, 11(4), p. 1165. Available at: https://doi.org/10.3390/ani11041165
