# Supplementary material for: Risk factors associated with Crimean-Congo hemorrhagic fever virus circulation among human, livestock and ticks in Mauritania through a one health retrospective study
Source: BMC Infect Dis. 2023 Nov 6;23:764. doi: 10.1186/s12879-023-08779-8 (PMC10626674; doi:10.1186/s12879-023-08779-8)
Supplement: Supplementary file 2 — Supplementary Material 2 [file 12879_2023_8779_MOESM2_ESM.docx]

Supplementary Table 2: Modeled resultants

| Category | Model | AIC | Explained Variance | Dxy | AUC |
| --- | --- | --- | --- | --- | --- |
| Large Ruminants | (1 \| Province/Location) | 253.1 | 12% | 0.61 | 0.80 |
|  | Age+Sex+Species+(1 \| Province/Location) | 247.64 | 18.4% | 0.65 | 0.82 |
|  | Age+Sex+Species+NDVI+Betweenness+Cattle Density+ (1 \| Province/Location) | 229.1 | 26.2% | 0.67 | 0.83 |
| Small Ruminants | (1 \| Province/Location) | 219 | 11.3% | 0.53 | 0.76 |
|  | Species+Age+(1 \| Province/Location) | 203.7 | 15.2% | 0.52 | 0.76 |
|  | Species+Age+Longitude+Distance+(1\| Province/Location) | 194.5 | 26.4% | 0.69 | 0.85 |
| Humans | (1 \| Province/Location) | 53 | 13.3% | 0.77 | 0.88 |
|  | Type+Age+(1 \| Province/Location) | 45.7 | 35.0% | 0.96 | 0.98 |
|  | Type+Age+Block+ (1 \| Province/Location) | 50.3 | 33.3% | 0.95 | 0.97 |
