# Supplementary material for: Risk factors associated with Crimean-Congo hemorrhagic fever virus circulation among human, livestock and ticks in Mauritania through a one health retrospective study
Source: BMC Infect Dis. 2023 Nov 6;23:764. doi: 10.1186/s12879-023-08779-8 (PMC10626674; doi:10.1186/s12879-023-08779-8)
Supplement: Supplementary file 3 — Supplementary Material 3 [file 12879_2023_8779_MOESM3_ESM.docx]

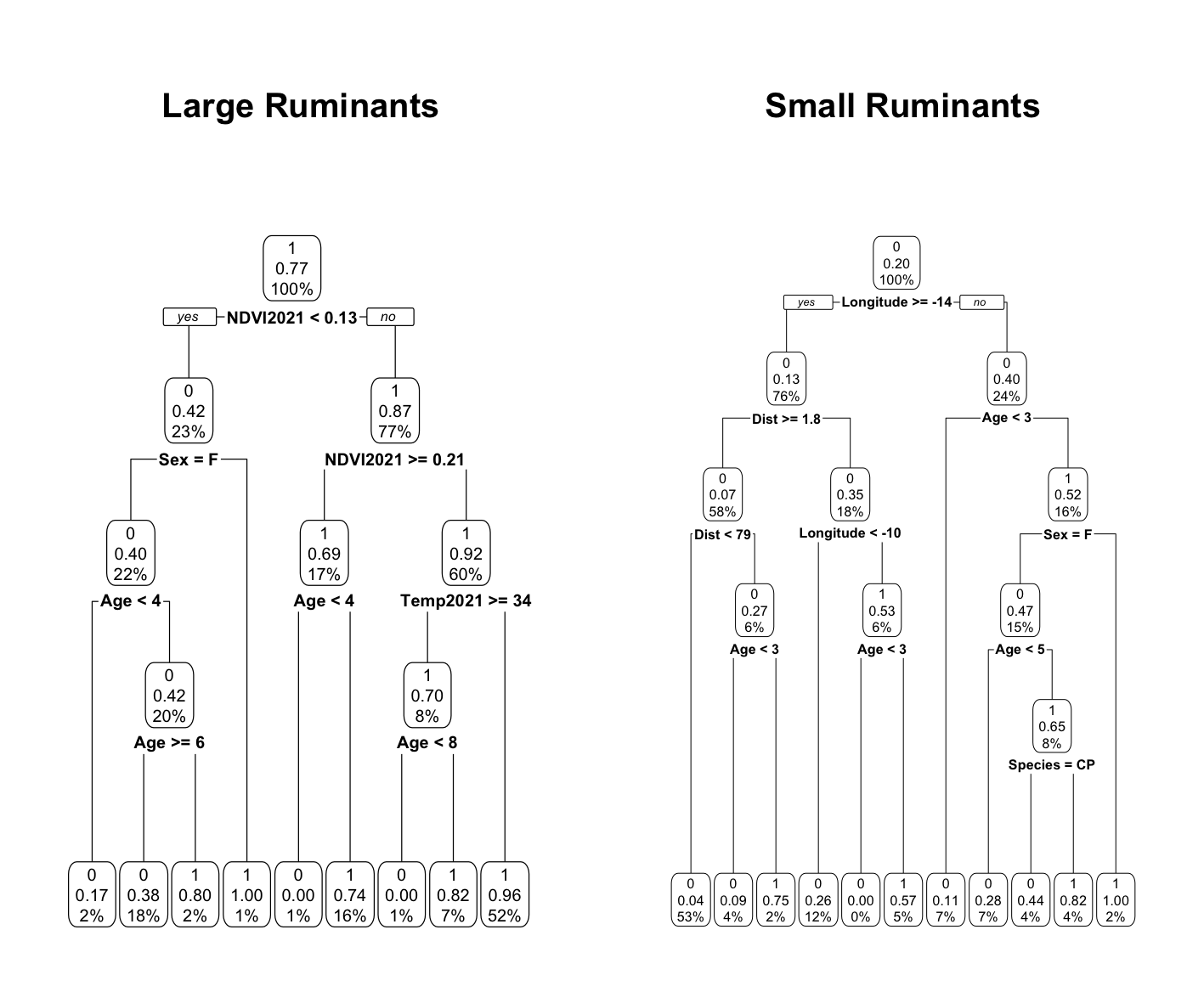


**Figure S1**

Fig S1 CARTs for large ruminants (left) and small ruminants (right).  For each category, the tree shows the different possible splitting rules that can be used to efficiently predict the type of result (seropositive or seronegative). Each box (decision node) corresponds to a single input predictor variable and a split threshold on that variable. The values in the box correspond to the average sero-positivity in the group and frequency  (%) after each cutoff.  The different cut-off values allow us to define groups that can be used for univariate analyses.
